# RNA Interference in Fungal Plant Pathogens: What Do We Know from *Botrytis cinerea* with Research Hotspots and Gaps, and What Are the Future Directions?

**DOI:** 10.3390/jof11070498

**Published:** 2025-07-01

**Authors:** Guy Smagghe

**Affiliations:** 1 Institute of Entomology, Guizhou University, Guiyang 550025, China; guysma9@gmail.com; 2 Department of Plants and Crops, Ghent University, 9000 Ghent, Belgium; 3 Department of Biology, Vrije Universiteit Brussel (VUB), 1050 Brussels, Belgium

**Keywords:** RNA interference, spray-induced gene silencing, *Botrytis cinerea*, gray mold, double stranded RNA, mechanisms of uptake, cell wall, nanoparticles, target genes, field trials, regulatory

## Abstract

RNA interference (RNAi) has emerged as a promising tool for controlling fungal plant pathogens, offering a targeted and environmentally friendly alternative to traditional chemical fungicides. *Botrytis cinerea*, the causative agent of gray mold disease, serves as a model and plant pathogen for investigating RNAi-based strategies due to its wide host range and economic impact. This review synthesizes current knowledge on RNAi mechanisms in *B. cinerea*, and that several factors influence the efficacy of RNAi in *B. cinerea*, including the stability and uptake of double-stranded RNAs (dsRNAs), the efficiency of RNA processing machinery, and environmental conditions. Furthermore, RNAi responses can vary significantly across strains, developmental stages, and infection modes, underscoring the complexity of fungal responses. With this review, I also aim to present the field trials reported so far, underscoring the practicality of RNAi. This review identifies current hotspots and outlines future directions for deploying RNAi as a sustainable control strategy against fungal pathogens.

## 1. Introduction

**Figure 1:**
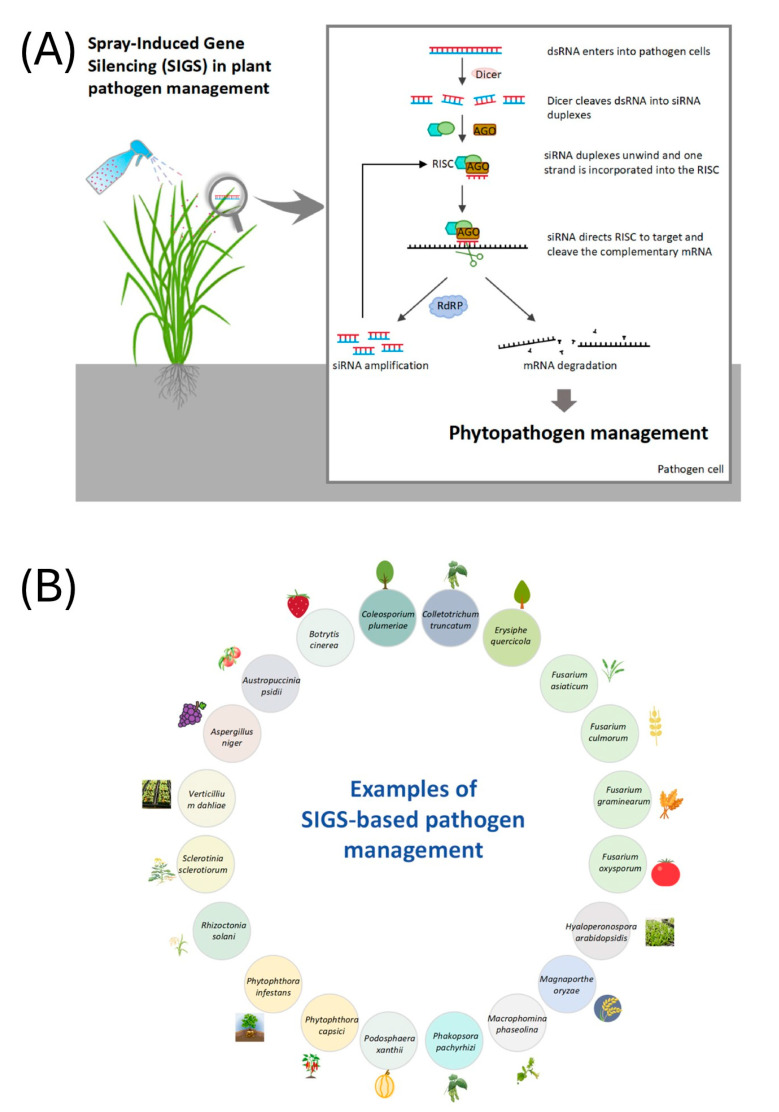
(**A**) The mechanism of spray-induced gene-silencing (SIGS)-based phytopathogen management, and (**B**) examples of SIGS-based pathogen management in plants. From He et al. [2].

RNA interference (RNAi) has emerged as a transformative approach in plant pathology, offering precise and environmentally sustainable methods for controlling plant pathogens. This natural gene-silencing mechanism operates by degrading specific messenger RNA (mRNA) molecules, thereby inhibiting the expression of genes essential for pathogen survival and virulence. By designing double-stranded RNA (dsRNA) molecules that correspond to critical genes in pathogens, researchers can trigger RNAi responses that suppress the growth and infectivity of fungi, bacteria, and viruses affecting plants. Recent advancements have highlighted the efficacy of RNAi-based strategies in managing diseases caused by important plant pathogens such as *Botrytis cinerea*. Techniques like spray-induced gene silencing (SIGS) enable the external application of dsRNA, leading to gene silencing in pathogens without the need for genetic modification of the host plant. This SIGS-based pathogen management has been tested in different fungal pathogens including *B. cinerea* (Figure 1). This approach not only provides a sustainable alternative to chemical pesticides but also minimizes environmental impact. As research progresses, RNAi holds significant promise for enhancing crop protection and ensuring food security (see [1] and references therein).

*B. cinerea*, the causative agent of grey mold, is a pervasive fungal pathogen that affects over 1400 plant species, including economically significant crops such as grapes, strawberries, tomatoes, and ornamental plants. Its capacity to infect a wide range of hosts, coupled with its ability to persist under various environmental conditions, makes it a formidable threat to agriculture [3,4,5]. To mitigate the impact of *B. cinerea*, fungicides have been extensively used. However, the pathogen’s high genetic variability and adaptability have led to the emergence of resistance against multiple fungicide classes, including benzimidazoles, dicarboximides, and quinone outside inhibitors. Notably, strains exhibiting multiple resistance (MLR) and multidrug resistance (MDR) have been identified, characterized by mutations in target genes and overexpression of efflux transporters, respectively. The proliferation of resistant *B. cinerea* strains underscores the need for integrated disease management strategies. These strategies include the rotation and combination of fungicides with different modes of action, implementation of cultural practices to reduce pathogen pressure, and exploration of alternative control measures such as biological agents and RNAi technologies [6,7,8,9]. In this paper, I have chosen *B. cinerea* as a model organism and important disease to present the current knowledge on RNAi, its mechanisms, influencing factors, variability, field testing, and optimization strategies. Finally, the current conclusions, along with research hotspots and gaps, are discussed with future perspectives.

## 2. Mechanisms of RNAi in *B. cinerea*

**Figure 2:**
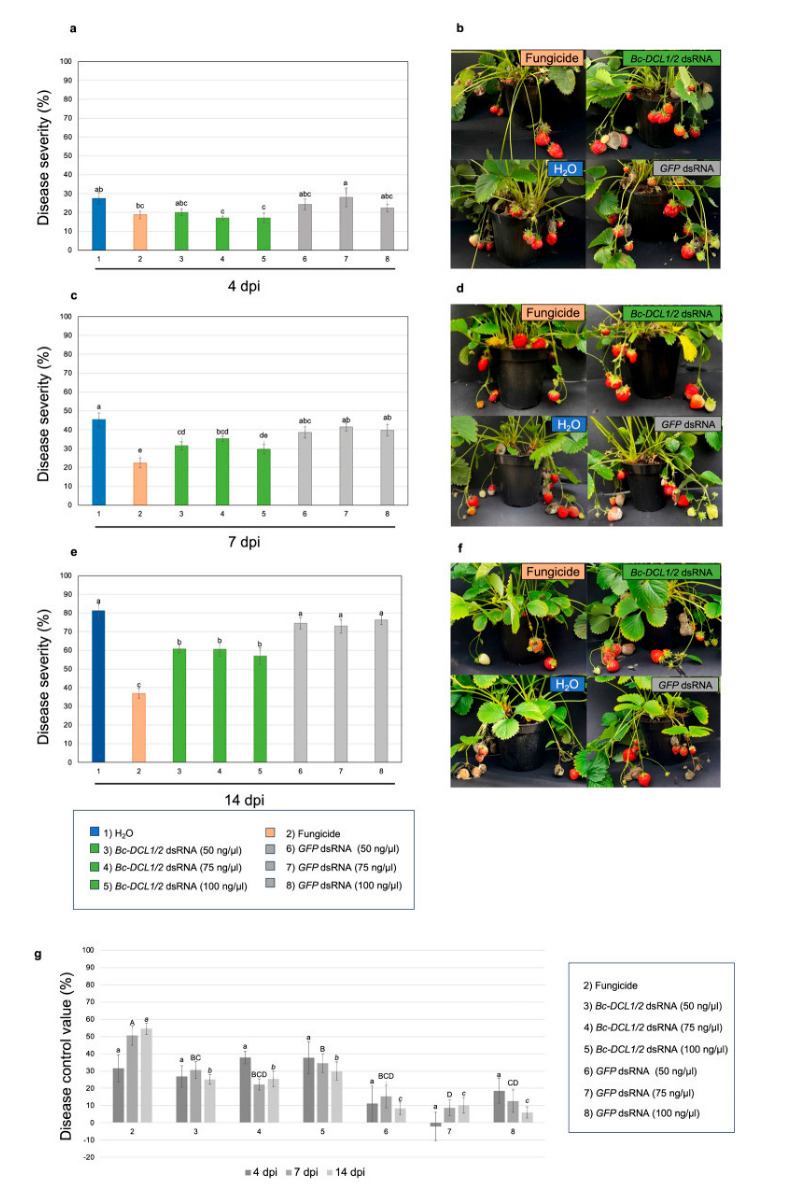
Effect of exogenous application of *Bc-DCL1/2* dsRNA molecules on plants of strawberry cultivated in the greenhouse for the control of *B. cinerea* disease. Disease severity observed after (**a**) 4, (**c**) 7, and (**e**) 14 dpi on plants treated with 50, 75, and 100 ng/μL of *Bc-DCL1/2* dsRNA or of GFP dsRNA molecules, compared with commercial fungicide and water treatments; representative photos of strawberry plants from each type of treatment after (**b**) 4, (**d**) 7, and (**f**) 14 dpi. (**g**) Disease control value (%) exerted by fungicide and dsRNA-based treatments of gray mold on strawberry fruits observed at 4, 7, and 14 days after *Botrytis* inoculation in the greenhouse. Values followed by small letter (a) compare data at 4 dpi, by capital letters (A, B, C, D) at 7 dpi, by italics small letters (*a*, *b*, *c*) at 14 dpi. Means with different letters are significantly different according to the Student Newman–Keuls test (*p*  ≤  0.05)  ±  SE. Error bars represent the standard errors of three replications. From Capriotti et al. [7].

**Figure 3:**
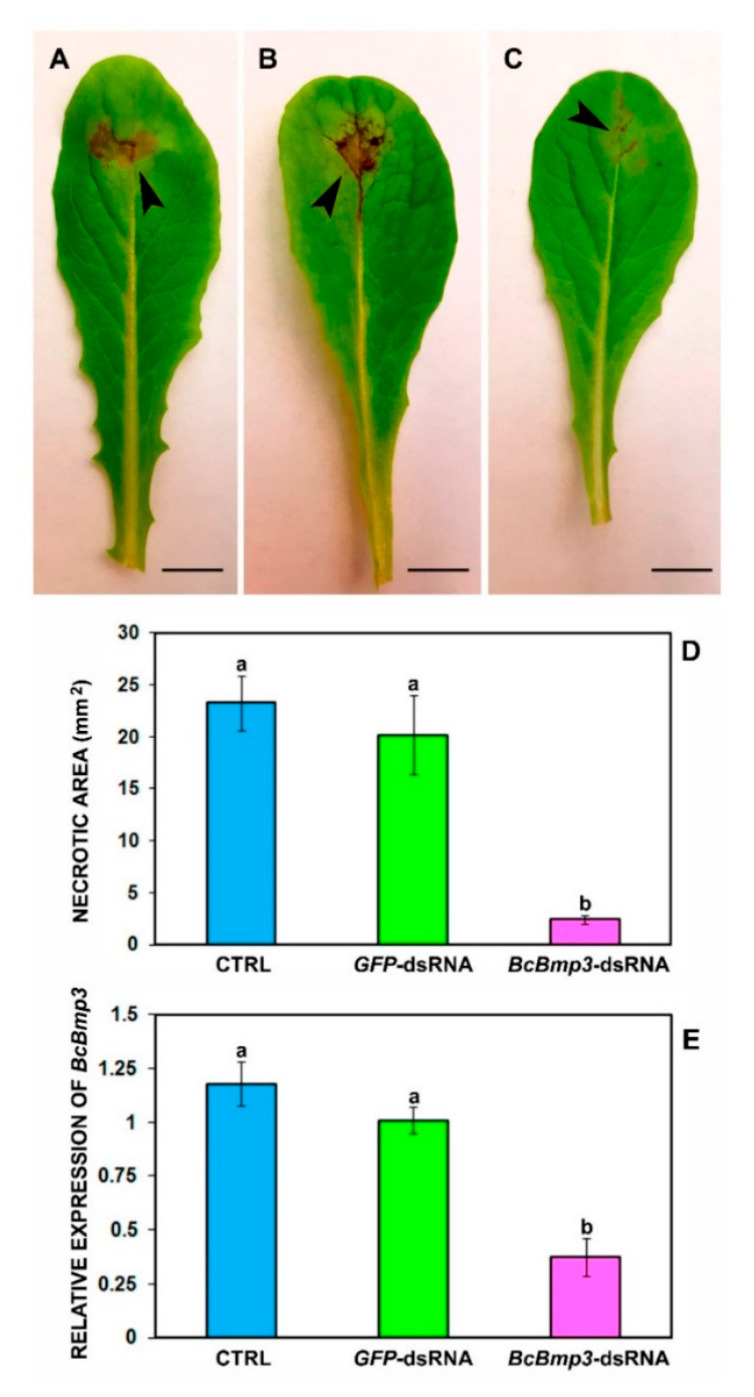
Infection symptoms of *B. cinerea* B05.10 on leaves of lettuce (*Lactuca sativa* cv. Romana) at 5 dpi. (**A**) Leaves were treated with water + TE (CTRL), (**B**) GFP-dsRNA, and (**C**) BcBmp3-dsRNA. Infection symptoms of *B. cinerea* B05.10 on leaves of *Lactuca sativa* cv. Romana at 5 dpi. (**A**) Leaves were treated with water + TE (CTRL), (**B**) GFP-dsRNA, and (**C**) BcBmp3-dsRNA, and then were artificially inoculated with 5 μL of a conidial suspension (500 conidia) of the pathogen. (**D**) The necrotic areas (in mm^2^) were measured. The graph shows the mean (±SE) values of two independent experiments with sixteen biological replicates (n = 16). (**E**) Transcription of BcBmp3 mRNA at 5 dpi. Relative transcript values were calculated by qRT-PCR using GFP-dsRNA and CTRL as reference samples and normalized to BctubA gene. The graph shows the mean (±SE) values of three biological replicates (n = 3). The same letters above the bars indicate no significant differences from each other (ANOVA) according to Tukey’s test (*p* 0.05). Scale bars = A: 8.8 mm; B: 8.9 mm; C: 8.4 mm. From Spade et al. [10].

As explained above, RNAi is a conserved biological process where dsRNA molecules inhibit gene expression by promoting the degradation of specific mRNA targets. In the context of *B. cinerea*, the exogenous application of dsRNA could silence essential fungal genes, thereby reducing virulence and pathogenicity. Key genes targeted include the following:

- Dicer-like genes (BcDCL1 and BcDCL2): These genes are integral to the fungal RNAi machinery. Figure 2 demonstrates that the silencing of them disrupts the production of small RNAs that *B. cinerea* uses to suppress host plant defenses, thereby attenuating fungal virulence [7].
- Mitogen-activated protein kinases (MAPKs) such as BcBmp3: These are involved in fungal growth, development, and pathogenicity. As shown in Figure 3, targeting BcBmp3 with dsRNA has been shown to significantly reduce fungal growth and lesion formation on host plants [8,10].

## 3. Factors Influencing RNAi Efficacy

**Figure 4:**
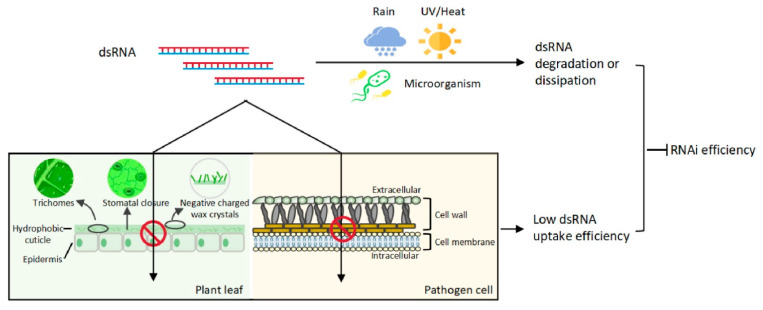
dsRNA stability and cellular uptake efficiency are two main factors affecting RNAi efficiency. Environmental factors such as rain, UV irradiation/heat, and microorganisms can lead to dsRNA degradation or dissipation. Also, leaf wettability determined by trichomes, stomata, hydrophobic cuticles, and wax crystals acts as a barrier to foliar uptake of sprayed dsRNA. In addition, cell walls and cell membranes of pathogens may hinder cellular uptake of dsRNA. The degraded/dissipated dsRNA or the low dsRNA uptake efficiency will contribute to the inhibition of RNAi efficiency. From He et al. [2].

Over the past years, different laboratories have studied several factors that affect the success of RNAi-based strategies against *B. cinerea*. In summary, three important factors are as follows:

- dsRNA stability: Naked dsRNA is prone to rapid degradation by environmental nucleases, limiting its effectiveness. To address this, formulations like BioClay™, which combines dsRNA with layered double hydroxide (LDH) clay particles, have been developed. This approach has been shown to significantly enhance dsRNA stability and prolong its protective activity. For instance, BioClay™ increased the protection window against *B. cinerea* from 1 to 3 weeks on tomato leaves and from 5 to 10 days on tomato fruits, compared to naked dsRNA [11,12,13,14,15]. Additionally, other nanocarriers, such as artificial nanovesicles and RNA nanoparticles, have been explored for dsRNA delivery. These carriers can protect dsRNA from degradation and facilitate its uptake by the fungus, thereby enhancing the RNAi effect [16,17]. Figure 4 presents that environmental factors such as rain, UV irradiation/heat, and microorganisms can lead to dsRNA degradation or dissipation. Furthermore, leaf wettability determined by trichomes, stomata, hydrophobic cuticles, and wax crystals acts as a barrier to foliar uptake of sprayed dsRNA. In addition, cell walls and cell membranes of pathogens may hinder cellular uptake of dsRNA. The degraded/dissipated dsRNA or the low dsRNA uptake efficiency will contribute to the inhibition of RNAi efficiency. Thus, dsRNA stability and cellular uptake efficiency are two main factors affecting RNAi efficiency [2].

- Delivery methods: The method of dsRNA application significantly influences its uptake by *B. cinerea*. SIGS is a prominent technique where dsRNA is sprayed onto plant surfaces, allowing the pathogen to absorb it during infection. Studies have demonstrated the efficacy of SIGS in reducing disease symptoms in various crops. For example, dsRNA targeting specific genes in *B. cinerea* reduced disease severity in lettuce plants [2,7,15]. Moreover, the use of nanocarriers in SIGS can improve dsRNA delivery efficiency. Nanoparticles can facilitate the penetration of dsRNA through plant cuticles and enhance its stability, leading to more effective gene silencing [12,14,17,18].
- Target gene selection: Selecting essential and conserved genes in *B. cinerea* is crucial for effective RNAi-based control. Genes involved in vital processes such as cell wall synthesis, signal transduction, and virulence are prime targets. For instance, targeting the Dicer-like genes (*BcDCL1* and *BcDCL2*), which are involved in the RNAi pathway of the fungus, has been shown to reduce its virulence [7]. Additionally, genes like *BcBmp1*, *BcBmp3*, and *BcPls1*, which play roles in pathogenicity, have been effectively targeted using dsRNA, resulting in decreased disease symptoms in host plants [7]. Interestingly, recent data of Jin et al. [19] demonstrated that BcTRE1-targeting dsRNA (BcTRE1-dsRNA) exhibited the highest inhibitory activity, significantly reducing fungal growth and lesion formation of *B. cinerea* compared to the other tested dsRNAs. Gene expression analysis confirmed that BcTRE1-dsRNA effectively silenced BcTRE1 expression within 7 days post-treatment, aligning with the transient protection window observed for naked dsRNA applications. To extend this protective period, these authors incorporated LDH nanocarriers for dsRNA delivery, which successfully prolonged the inhibitory effect, reducing lesion formation even at 11 days post-treatment. These findings identify BcTRE1 as a key RNAi target and highlight the potential of dsRNA-LDH formulations for sustainable fungal disease management in crops.

By focusing on these three critical factors, including dsRNA stability, delivery methods, and target gene selection, researchers can enhance the effectiveness of RNAi-based strategies against *B. cinerea*. Continued advancements in nanotechnology and molecular biology are expected to further improve these approaches, offering sustainable solutions for crop protection [12,17,18].

## 4. Variability in RNAi Responses

The effectiveness of RNAi strategies against *B. cinerea* can indeed vary due to several factors. Recent studies have highlighted the following key aspects:

- Genetic diversity of *B. cinerea*: *B. cinerea* exhibits significant genetic variability, which can influence the efficacy of RNAi-based approaches. Variations in target gene sequences among different strains may affect the binding efficiency of dsRNA molecules, leading to inconsistent gene-silencing outcomes. For instance, Qin et al. [20] reported that certain isolates, known as “vacuma” strains, lack active transposons and have been reported to be less virulent on specific hosts. This genetic diversity necessitates careful selection and validation of target genes for RNAi applications.
- Environmental conditions: Environmental factors such as temperature, humidity, and ultraviolet (UV) exposure can significantly impact the stability and uptake of dsRNA molecules. For example, UV radiation can degrade dsRNA, reducing its availability for uptake by the pathogen. Additionally, environmental conditions can influence the efficiency of dsRNA uptake mechanisms in fungi. Studies have shown that *B. cinerea* is capable of absorbing external dsRNA, but the efficiency of this process can vary depending on environmental conditions [21].
- Plant species and tissue specificity: The interaction between *B. cinerea* and its host plant can differ among plant species and even among different tissues within the same plant. These differences can affect the success of RNAi strategies. For instance, the uptake and systemic movement of dsRNA can vary between plant species, influencing the availability of dsRNA to the pathogen. Moreover, the structural and biochemical properties of different plant tissues can affect dsRNA stability and uptake. Therefore, tailoring RNAi approaches to specific plant–pathogen interactions is crucial for effective disease control [7].

Understanding these factors is essential for optimizing RNAi-based strategies against *B. cinerea*. By considering the genetic diversity of the pathogen, environmental conditions, and host plant characteristics, researchers can enhance the efficacy of RNAi applications in plant disease management.

## 5. Field Trials and Efficacy of RNAi to Control *B. cinerea*

**Figure 5:**
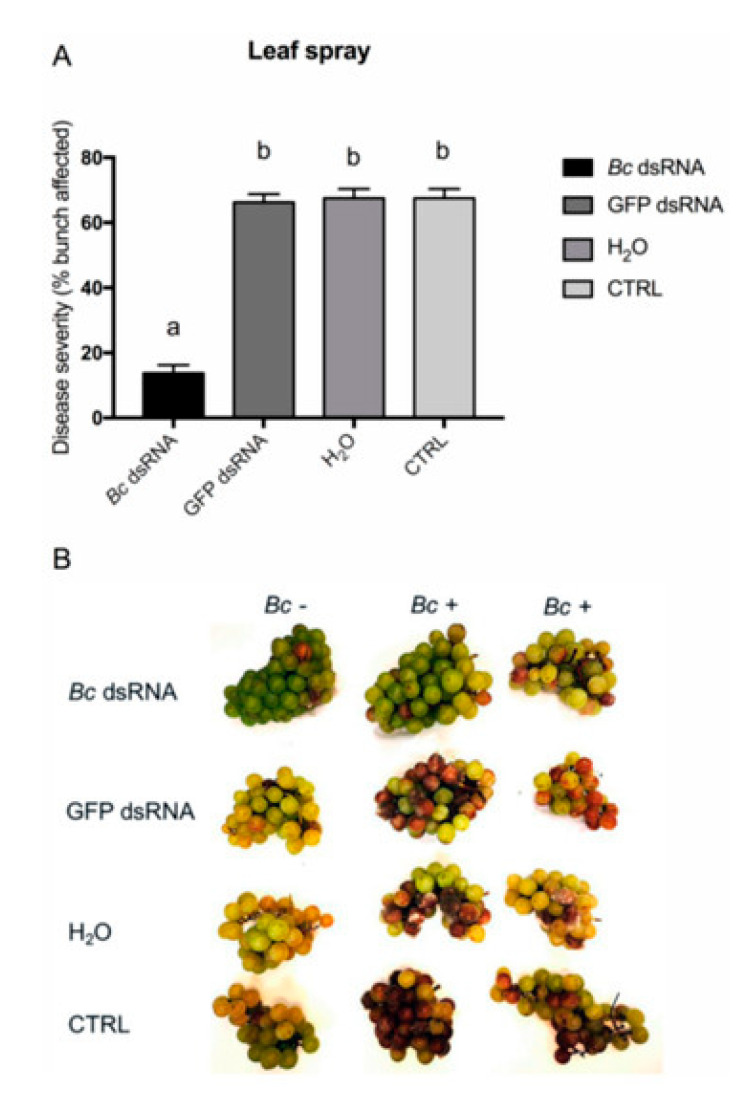
Disease severity on bunches recorded for the in vivo high-pressure leaf spray application method. (**A**) Percentage of berries affected following *Bc* dsRNA, GFP dsRNA, water (H_2_O) application, and in untreated CTRL samples. Different lowercase letters above bars indicate significant differences as determined by Tukey’s HSD test (*p* ≤ 0.05) (**B**). Overview of representative samples for each condition tested. One bunch was maintained as uninoculated control (*Bc* −) and two inoculated (*Bc* +) bunches of cv. Moscato are shown. From Nerva et al. [27].

The first experiments utilizing RNAi in *B. cinerea* were reported by Patel et al. [22] nearly two decades ago. In their study, they successfully silenced a superoxide dismutase gene, demonstrating the feasibility of RNAi as a genetic manipulation tool in this fungal pathogen [23]. Since then, multiple laboratory studies have demonstrated the potential of RNAi in controlling *B. cinerea*. For instance, Wang et al. [24] applied small RNAs or dsRNAs targeting the Dicer-like genes BcDCL1 and BcDCL2 directly onto plant tissues, including strawberry fruits, which significantly inhibited gray mold disease. These findings underscore the promise of RNAi-based strategies in managing *B. cinerea* infections [7,25]. Following these laboratory assays, recent field and greenhouse studies have demonstrated the practical potential of RNAi-based strategies to control *B. cinerea* across various crops. Below is a summary of key findings:

- Strawberries. In greenhouse experiments, topical applications of dsRNAs targeting the *BcDCL1* and *BcDCL2* genes of *B. cinerea* were administered to strawberry plants. Capriotti et al. [7] report that the treatments led to a significant reduction in fungal susceptibility compared to untreated controls (Figure 2). However, the efficacy was somewhat lower than that achieved with chemical fungicides. These results underscore the potential of RNAi-based approaches for gray mold management in strawberries, while also highlighting the need for improved formulations to enhance dsRNA stability and longevity.
- Lettuce. Field trials on lettuce involved the application of dsRNAs targeting the *BcBmp1*, *BcBmp3*, and *BcPls1* genes of *B. cinerea* using SIGS. When combined with nanocarriers like synthetic LDH (sLDH) clay nanosheets, these treatments effectively reduced gray mold symptoms (Figure 3). The use of sLDH nanocarriers not only protected the dsRNA from environmental degradation but also facilitated controlled release, enhancing the duration of protection [26].
- Grapevines. Field experiments on grapevines demonstrated that dsRNA applications could control both pre- and post-harvest gray mold infections. Various delivery methods were tested, including high-pressure spraying of leaves, petiole adsorption, and post-harvest spraying of grape bunches. All methods effectively reduced fungal virulence, with post-harvest spraying showing particularly promising results. For instance, Figure 5 shows a significant decrease in disease severity on bunches recorded for the in vivo high-pressure leaf spray application method with *Bc* dsRNA. These findings highlight the applicability of RNAi-based strategies in fruit crops susceptible to *B. cinerea* [27].

In summary, these studies collectively underscore the potential of RNAi-based approaches as sustainable alternatives to conventional fungicides for managing *B. cinerea* infections. However, challenges such as dsRNA stability under field conditions and the development of efficient delivery systems remain to be addressed to fully realize the practical applications of this technology.

## 6. Optimization Strategies

Building upon the previously discussed factors affecting the efficacy of RNAi strategies against *B. cinerea*, recent research has proposed several optimization strategies to enhance their effectiveness and practicality [15,28,29,30,31]:

- Nanoparticle-based delivery systems: Incorporating dsRNA into nanoparticles can protect it from environmental degradation and facilitate controlled release, thereby extending the duration of protection. For instance, Niño-Sánchez et al. [32] reported on LDH nanoparticles, such as those used in BioClay™, and these have been shown to enhance dsRNA stability and prolong its protective effects against *B. cinerea* infections in crops like tomatoes. Similarly, chitosan-based nanoparticles have demonstrated potential in shielding dsRNA and improving its delivery efficiency.
- Combination therapies: Employing RNAi in conjunction with other control methods can enhance overall effectiveness and mitigate resistance development. Integrating RNAi with biological control agents (BCAs), such as microbial biofungicides, or applying reduced doses of chemical fungicides has shown promise in managing *B. cinerea* infections. This integrated approach can lead to synergistic effects, improving disease control while reducing reliance on chemical inputs.
- Targeting multiple genes: Designing dsRNA molecules that simultaneously target multiple essential genes in *B. cinerea* can increase the likelihood of successful gene silencing and reduce the risk of resistance development. For example, targeting both Dicer-like genes (*BcDCL1* and *BcDCL2*), which are crucial for the fungus’s RNAi machinery, has been effective in compromising its virulence. Additionally, RNA nanostructures capable of delivering multiple small interfering RNAs (siRNAs) have been developed to enhance the breadth and durability of RNAi responses [13,33,34].
- Advancements in dsRNA production: Developing cost-effective and scalable methods for dsRNA synthesis is vital for the widespread adoption of RNAi-based strategies. Utilizing microbial biofactories, such as genetically engineered bacteria, to produce dsRNA offers a sustainable and economical approach. These microbial systems can be harnessed to generate large quantities of dsRNA targeting specific fungal genes, facilitating practical field applications [35].

By implementing these optimization strategies, researchers and agricultural practitioners can enhance the efficacy and practicality of RNAi-based control methods against *B. cinerea*, contributing to more sustainable and effective plant disease management.

## 7. Conclusions: Hotspots, Gaps, and Future Directions

It can be concluded that RNAi presents a promising, sustainable approach to managing *Botrytis cinerea* infections in agriculture. By understanding and optimizing the mechanisms, delivery methods, and target selection, RNAi-based strategies can be effectively integrated into crop protection programs, reducing reliance on chemical fungicides and mitigating environmental impacts. The current research hotspots and gaps are listed below. Evidently, an understanding of these cross-kingdom RNAi mechanisms is crucial for developing innovative strategies to enhance crop resistance against pathogens like *B. cinerea*.

- SIGS: This technique involves the topical application of dsRNA to plants, leading to gene silencing in pathogens upon contact. SIGS has shown promise against *B. cinerea*, offering a non-transgenic method for disease control.
- Nanocarrier systems: To enhance the stability and delivery of dsRNA, researchers are exploring nanotechnology-based carriers, such as artificial nanovesicles and LDHs. These systems protect dsRNA from environmental degradation and facilitate its uptake by fungal pathogens.
- Cross-kingdom RNAi mechanisms: Cross-kingdom RNAi is a bidirectional communication mechanism wherein small RNAs (sRNAs) are exchanged between plants and pathogens like *Botrytis cinerea*, leading to gene silencing across species boundaries [21,33]. Understanding how plants and pathogens exchange small RNAs is crucial. Studies have shown that pathogens like *B. cinerea* can deliver small RNAs into plant cells to suppress immunity, while plants can reciprocate by sending silencing RNAs into the pathogen [18]. In detail, *B. cinerea* secretes sRNAs that can enter plant cells and suppress host immunity. These fungal sRNAs are packaged into extracellular vesicles (EVs) and delivered into plant cells via clathrin-mediated endocytosis. Once inside, they hijack the plant’s Argonaute 1 (AGO1) protein to silence defense-related genes, facilitating infection [29,34,36]. Conversely, plants can produce sRNAs that target and silence genes in *B. cinerea*. These plant-derived sRNAs are also delivered via EVs into the fungal cells, where they can suppress fungal virulence genes, enhancing plant resistance. Recently, Cheng et al. [37] revealed that members of fungal plant pathogen *B. cinerea* BcAGO family contribute to plant infection. Specifically, BcAGO1 binds to both fungal and plant small RNAs during infection and acts in bidirectional cross-kingdom RNAi, from fungus to plant and vice versa. BcAGO2 also binds fungal and plant small RNAs but acts independent from BcAGO1 by regulating distinct genes. Nevertheless, BcAGO2 is important for infection, as it is required for effective pathogen small RNA delivery into host cells and fungal induced cross-kingdom RNAi. Providing these mechanistic insights of pathogen AGOs promises to improve RNAi-based crop protection strategies.

Building upon the previously discussed factors affecting the efficacy of RNAi strategies against *B. cinerea*, recent research has identified several critical gaps and future directions that need to be addressed to optimize these approaches:

- Mechanistic studies in different fungi: While the uptake of dsRNA by fungi like *B. cinerea* has been observed, the specific cellular and molecular mechanisms facilitating this process remain incompletely understood, especially in other fungi than *B. cinerea*. Studies have shown that *B. cinerea* can internalize exogenous dsRNA, leading to gene silencing; however, the pathways and proteins involved in dsRNA uptake are not fully elucidated. Further research into the cellular uptake pathways of dsRNA in *B. cinerea* and also in other fungal pathogens will aid in optimizing delivery methods. Understanding these mechanisms is crucial for enhancing the efficiency of RNAi-based control methods against fungal plant pathogens. Indeed, addressing these research gaps through targeted studies and policy development will be pivotal in advancing RNAi-based strategies for sustainable crop protection against important plant pathogens like *B. cinerea*.
- Environmental stability: Ensuring the stability of dsRNA under field conditions is a significant challenge. Unformulated dsRNA is susceptible to rapid degradation due to environmental factors such as UV radiation, microbial activity, and soil composition. To address this, researchers are exploring protective delivery systems, including nanocarriers like chitosan and LDH nanoparticles, which can shield dsRNA from degradation and facilitate controlled release, thereby extending its protective effects [31,38].
- Field trials: Conducting large-scale field trials will be essential to assess the real-world efficacy and environmental impact of RNAi-based fungicides. While laboratory studies have demonstrated the potential of RNAi strategies, their performance under field conditions remains to be thoroughly evaluated. Field trials will provide valuable data on factors such as dsRNA stability, delivery efficiency, and overall effectiveness in diverse environmental settings. In parallel, resistance development can be followed, similar to the proactive management strategy as proposed for RNAi-based control of pest insects [39].
- Integration into integrated pest management (IPM): Incorporating RNAi strategies into IPM programs can provide a holistic approach to disease control. RNAi-based applications, such as SIGS, offer species-specific targeting of pathogens with minimal impact on non-target organisms (NTOs). By integrating RNAi with other control methods, such as biological control agents and cultural practices, it is possible to enhance overall pest management efficacy and sustainability.
- Regulatory frameworks: The deployment of RNA-based fungicides necessitates comprehensive regulatory guidelines to assess their safety and efficacy. Establishing clear regulatory guidelines is vital to facilitate the adoption of RNA-based plant protection products. Currently, regulatory frameworks for RNAi-based biopesticides are still evolving. In the European Union, these products are subject to a two-step approval process involving the European Food Safety Authority (EFSA) and individual Member States. In the United States, the Environmental Protection Agency (EPA) evaluates RNAi-based biopesticides as biochemical pesticides, requiring data to demonstrate their safety for humans and the environment. Developing clear and harmonized regulatory policies is essential to facilitate the adoption of RNAi technologies in agriculture [40,41,42].
